# Optic coherence tomography findings in relapsing-remitting multiple sclerosis patients of the northwest of Iran

**Published:** 2013

**Authors:** Mahnaz Talebi, Masoud Nikanfar, Rana Sorkhabi, Ehsan Sharifipour, Mansour Bahrebar, Ali Kiavar, Sasan Andalib, Hadi Mohammad Khanli

**Affiliations:** 1 Associate Professor, Department of Neurology AND Neurosciences Research Center (NSRC), Tabriz University of Medical Sciences, Tabriz, Iran; 2 Associate Professor, Department of Ophthalmology, School of Medicine, Tabriz University of Medical Sciences, Tabriz, Iran; 3 Resident, Department of Neurology AND Neurosciences Research Center (NSRC), Student Research Committee, Tabriz University of Medical Sciences, Imam Reza Hospital, Tabriz, Iran; 4 Resident, Department of Ophthalmology, School of Medicine, Tabriz University of Medical Sciences, Tabriz, Iran; 5 PhD Student, Neurosciences Research Center (NSRC), Tabriz University of Medical Sciences, Tabriz, Iran; 6 Neurologist, Neurosciences Research Center (NSRC), Tabriz, Iran

**Keywords:** Relapsing-Remitting Multiple Sclerosis, Optic Coherence Tomography, Optic Neuritis

## Abstract

**Background:**

Optical coherence tomography (OCT) is a simple, high-resolution technique to quantify the thickness of retinal nerve fiber layer (RNFL) and macula volume, which provide an indirect measurement of axonal damage in multiple sclerosis (MS). This study aimed to evaluate OCT finding in relapsing-remitting MS patients of the northwest of Iran and compare them with a normal control group.

**Methods:**

In a cross-sectional, descriptive, analytic study, 60 patients with MS as case group and 60 patients as controls were studied. Total macular volume (TMV) and retinal nerve fiber layer (RNFL) in perioptic disk area (3.4 millimeter around the disk) and macula was measured using Stratus 3000 in circular form. These findings were compared between the two groups and their relationship with the duration and severity of MS [based on Expanded Disability Status Scale (EDSS)] and history of optic neuritis were evaluated.

**Results:**

In total, 35 men and 85 women with a mean age of 34.8 years were evaluated. The mean RNFL in MS patients were 231.9 and 233.1 micrometers in right and left eyes; while they were 246.7 and 250.4 micrometers in right and left eyes of healthy subjects, respectively. This difference in thickness of RNFL in total measure and all quadrants around the optic disk and TMV between case and control groups was analytically meaningful (P = 0.001 and P = 0.001 for right and left eyes, respectively). The mean thickness of RNFL in patients with optic neuritis was significantly lower than other patients in right and left eyes (P = 0.042 and P = 0.005). There was a significant correlation between most of OCT findings and the MS disease duration and EDSS.

**Conclusion:**

Findings of the present study in the northwest of Iran buttress the idea that RNFL thickness can be greatly affected by MS. Our results also indicate that this effect is associated with ON and MS duration and severity.

## Introduction

Multiple sclerosis (MS) refers to a chronic inflammatory disease of the central nervous system. It includes an immune-based inflammation characterized by demyelination of nerve fibers leading eventually to axonal damage.^1, 2^ In the development of MS, 30 to 70% of patients present with acute optic neuritis (ON); even so, 94 to 99% of them show plaques in optic nerve in post-mortem assessment.^3, 4^

Axons of demyelinated neurons are attractive sites for evaluation of axonal loss. On this account, retinal nerve fiber layer (RNFL), which is the innermost layer of the retina, is the best target for this assessment. The most plausible explanation is that the layer is formed by retinal ganglion cell axons myelinated after they pass through the lamina cribrosa.^5^ Optic nerve demyelination (either in clinical form or in subclinical form) appears as a retrograde degeneration of optic nerve axons giving rise to a thinner retinal nerve fiber layer (RNFL).^6^ Diminished RNFL thickness has been demonstrated in MS. Assessment of RNFL thickness, hence, is a good marker for axonal damage in patients with MS.^5^

Special attention has recently been devoted to optical coherence tomography (OCT) as a valuable tool in neurological assessments mostly used for neuro-ophthalmological involvements.^7–10^ OCT is a simple, accurate, non-invasive, and rapid diagnostic apparatus providing high-resolution, cross-sectional images of the retina, and it can be performed in office-based settings. By means of OCT, RNFL thickness around optic disc and total macular volume (TMV) can be quantified.^11–13^ Pathological specificity, appropriate reproducibility and reliability, and the ability to show RNFL and TMV changes on a time scale are some of the characteristics of OCT.^14–17^ The present study set out to investigate OCT findings pertaining to RNFL in relapsing-remitting MS patients, and assess the association of disease duration and severity with optic neuritis and compare these findings with a normal population in the northwest of Iran.

## Materials and Methods

An analytical, observational, cross-sectional study design was applied for the present study. The study was carried out from May 2011 to October 2012 for a period of 15 months. 60 patients with relapsing-remitting (RR) MS and 60 healthy controls were assigned to case and control groups, respectively. Relapsing-remitting MS was diagnosed predicated upon the 2005 McDonald criteria. Inclusion criteria were defined as follows: 1) spherical equivalent of approximately ± 3 diopters; 2) signal strength of ≥ 7; and 3) vision correction of 0.5 diopters or less. Patients were excluded if: 1) they were in acute phase of acute optic neuritis; 2) they had a history of systematic and visual diseases; and 3) they had noticeable age-based visual change (macula-based degeneration). All the patients were under treatment of approved first choice medications; namely β interferons and Glatiramer acetate. No change was made in the administration of these medications. MS severity [assessed based upon Expanded Disability Status Scale (EDSS)], disease duration, optic neuritis (ON) history, and the number of relapses were recorded. Data pertaining to the RNFL thickness in superior, inferior, nasal, and temporal quadrants were compared between the case and control groups, and its association with disease duration, disease severity, and ON was evaluated. Analyzed variables consisted of gender, age, disease duration, ON history, EDSS, the number of relapses, macular volume, intraocular pressure, cup to disk ratio (C/D ratio) in left eye (OD) and right eye (OS), total RNFL thickness, superior RNFL thickness, inferior RNFL thickness, nasal RNFL thickness, temporal RNFL thickness, and total macular volume (TMV). A written informed consent was obtained from each participant. The present study was approved by the ethical committee of Tabriz University of Medical Sciences, Iran.

### Ophthalmologic evaluation

Having recorded the patients’ history with respect to systemic disease, visual problems, and consumed systemic and local eye medications; we evaluated various parts of eyes such as cornea, anterior chamber, lens, anterior vitreous by a slit lamp. In the case group, intraocular pressure was evaluated by Goldman tonometry. Afterwards, the retina was assessed through dilated pupil using slit lamp and 90 nm lens, and indirect ophthalmoscopy with 20 nm lens. RNFL thickness was evaluated around the disk in the case and control groups using Stratus OCT 3000 (Carl Zeiss Meditec, Inc., Dublin, CA, USA). Circular map was obtained by means of Fast RNFL 3.4 and Fast Macula Thickness Map protocols (software: Retinal map analysis report 4.0.5) from 3.4 mm around the disk in the macula region. For TMV assessment, six radial lines centered on the macula were obtained. A suitable quality of images was defined as follows: generalized signal distribution, a reflectance signal from either RNFL or retinal pigment epithelium strong enough to identify either layer, no missing parts caused by eye movements, and a signal strength of ≥ 7 of 10.^18^ In OCT, depending on reflection intensity, reflected light from retina is recorded as warm colors (white and red) with high reflection and cold colors (blue and black) with low reflection with a variation of approximately 10 microns. In RNFL topography, two peaks appeared around the disk on the inferior and superior RNFL. Imaging termed “TSINT Plot” initiated from temporal quadrant, continued on anterior, nasal, and posterior quadrants and finally reached temporal quadrant.

### Statistical Analysis

Using MedCalc Statistical Software (version 11.5.0, Brunswick, United States) and results of previously published studies, a sample size of 56 patients was calculated for each group. In order to increase the validity of the study and owing to the probability of sample loss, 60 patients (120 eyes) were finally recruited for each group. The results were defined as mean ± standard deviation (mean ± SD), frequency, and percentage. Statistical analysis was performed using the SPSS for Windows (version 16.0; SPSS Inc., Chicago, IL., USA). More precisely, data were analyzed by Student's t-test (for quantitative variables), and chi-square and Fisher's exact tests (for categorical variables). The Pearson correlation was applied for the assessment of the association between OCT findings and disease severity and duration. A P-value < 0.05 was considered significant.

## Results

In the present study, 35 subjects (29.2%) were male and 85 female (70.8%). Table 1 provides a breakdown pertaining to distribution of demographic variables in the two groups. Of the 60 patients with RRMS, 35 cases (58.3%) suffered ON. Right and left eye involvement was seen in 35 (58.3%) and 19 (54.3%) cases, respectively. Nonetheless, in 9 cases (20%) both eyes were involved. The EDSS mean in the MS patients was 2.5 ± 1.6 ranging from 0 to 7.5. The mean for the number of relapses in patients was 2.58 ± 1.74 times ranging from 0 to 6 times. Table 1 depicts the findings with regard to intraocular pressure, RNFL thickness, and TMV in both eyes and a comparison of these data between the case and control groups. RNFL thickness in all patients was found to be significantly diminished in all quadrants. Moreover, TMV of both eyes in all patients was significantly less than the normal group. RNFL in the MS patients with a history of ON was thinner in all quadrants, in comparison with patients without ON (Table 2). The mean for disease duration was 3.9 ± 5.8 years ranging from 1 to 16 years. Disease duration was observed to be associated with TMV in right eyes (P = 0.023; r = -0.294), TMV in left eyes (P = 0.013; r = -0.319), RNFL in right eyes (P = 0.000; r = -0.415), and RNFL in left eyes (P = 0.000; r = -0.423). More precisely, disease duration was significantly associated with the thickness of RNFL in all quadrants in both eyes (Table 3). 26 (43.3%), 24 (40%), 8 (13.3%), and 2 (3.4%) cases were shown to experience EDSS of 0-2, 2-4, 4-6, and 6-8, respectively. No patient was seen to show an EDSS of more than 8. Amongst the OCT findings in the present study, RNFL thickness in all quadrants in the left eyes, and the nasal and temporal quadrants in the right eyes were significantly associated with EDSS (Table 3). Furthermore, a significant association was found between the mean for TMV and disease severity (EDSS) in the left and right eyes.

**Table 1:** Comparison of demographic and measured variables♦

Characteristics	MS patients (Cases) * 60 (50.0%)	Normal (Control) * 60 (50.0%)	P
Gender			
Male	15 (25.0%)	20 (33.3%)	0.210
Female	45 (75.0%)	40 (66.7%)	
Age (years)	34.8 ± 8.4 (22-54)	33.5 ± 5.3 (21-42)	0.110
Intra optic pressure (mmHg):	14.3 ± 1 (13-17)	13.9 ± 1.5 (11-17)	0.056
Cup/Disk ratio*	0.34 ± 0.05 (0.3-0.5)	0.27 ± 0.04 (0.2-0.3)	0.001
OD RNFL total*	88.25 ± 19.56 (45-145)	100.7 ± 19.71 (45-158)	0.001
OD RNFL inferior*	108.15 ± 20.41 (51-138)	128.76 ± 14.47 (104-56)	0.001
OD RNFL superior*	114.04 ± 19.89 (47-145)	130.26 ± 14.94 (101-58)	0.001
OD RNFL nasal*	63.31 ± 14.96 (45-121)	70.76 ± 8.20 (45-82)	0.001
OD RNFL temporal*	67.51 ± 16.14 (45-119)	70.50 ± 10.95 (54-101)	0.001
OS RNFL total*	87.75 ± 18.98 (45-144)	99.51 ± 27.54 (54-155)	0.001
OS RNFL inferior*	113.06 ± 19.96 (52-144)	130.13 ± 11.72 (107-55)	0.001
OS RNFL superior*	109.52 ± 18.90 (58-140)	127.73 ± 9.61 (106-143)	0.001
OS RNFL nasal*	62.30 ± 15.38 (45-114)	71.83 ± 10.49 (59-90)	0.001
OS RNFL temporal*	66.15 ± 16.62 (45-120)	68.36 ± 8.83 (54-94)	0.001
OD TMV**	6.23 ± 0.7 (4.2-7.5)	6.60 ± 0.6 (5-7.5)	0.025
OS TMV**	6.29 ± 0.7 (4.4-7.9)	6.52 ± 0.7 (4.5-7.9)	0.016
Disease duration ( years)	5.8 ± 3.9 (1-16)	-	
EDSS	2.5 ± 1.6 (0-7.5)	-	
Number of relapses	2.58 ± 1.74 (0-6)	-	

♦ Data are presented as mean ± SD

OS: Oculus Sinister (the left eye)

MS: Multiple Sclerosis

TMV: Total Macular Volume

** All measurements are in mm3

OD: Oculus Dexter (the right eye)

RNFL: Retinal Nerve Fiber Layer

EDSS: Expanded Disability Status Scale

* All measurements are shown in µm

**Table 2:** Comparison of OCT findings in patients with and without history of optic neuritis *

RNFL thickness*	ON +	ON −	P
OD RNFL total	83.01 ± 19.49	93.01 ± 17.12	0.042
OS RNFL total	81.81 ± 18.73	91.96 ± 16.74	0.005
OD RNFL inferior	101.42 ± 20	112.20 ± 17.31	0.002
OS RNFL inferior	104.37 ± 18.91	116.70 ± 17.03	0.001
OD RNFL superior	107.40 ± 19.30	118.36 ± 17.02	0.002
OS RNFL superior	102.54 ± 18.69	112.04 ± 16	0.003
OD RNFL nasal	60.11 ± 15.12	67.8 ± 13.79	0.004
OS RNFL nasal	58.74 ± 13.98	66.64 ± 16.33	0.001
OD RNFL temporal	63.11 ± 16.10	73.68 ± 14.33	0.005
OS RNFL temporal	61.62 ± 15.17	72.48 ± 16.78	0.008

Data are presented as mean ± SD.

OS: Oculus Sinister (the left eye)

RNFL: Retinal Nerve Fiber Layer

* All measurements are shown in µm.

OD: Oculus Dexter (the right eye)

ON: Optic Neuritis

TMV: Total Macular Volume.

**Table 3:** Correlation of OCT findings in patients with disease duration and severity

OCT Findings	MS disease duration		MS disease severity (EDSS)	

RNFL Thickness:*	r	P	r	P
OD RNFL total	0.415	0.001	0.278	0.037
OS RNFL total	0.422	0.001	0.288	0.027
OD RNFL inferior	0.403	0.001	0.238	0.069
OS RNFL inferior	0.418	0.001	0.262	0.043
OD RNFL superior	0.410	0.001	0.241	0.066
OS RNFL superior	0.415	0.001	0.285	0.027
OD RNFL nasal	0.437	0.001	0.297	0.023
OS RNFL nasal	0.425	0.001	0.277	0.037
OD RNFL temporal	0.430	0.001	0.306	0.024
OS RNFL temporal	0.428	0.001	0.293	0.025
TMV:**				
OD TMV	0.294	0.023	0.261	0.043
OS TMV	0.319	0.013	0.273	0.039

Data are presented as mean ± SD.

OS: Oculus Sinister (the left eye)

RNFL: Retinal Nerve Fiber Layer

TMV: Total Macular Volume

r: Pearson's correlation coefficient

** All measurements are shown in mm3

OD: Oculus Dexter (the right eye)

OCT: Optical Coherence Tomography

MS: Multiple Sclerosis

EDSS: Expanded Disability Status Scale

* All measurements are shown in µm.

## Discussion

In the current study, OCT was assessed in a homogenous population of MS patients and healthy controls in the northwest of Iran. Patients presenting with MS showed striking differences in RNFL thickness and TMV compared to healthy people. According to our study, OCT findings were associated with disease duration and severity. Moreover, we found that ON diminished RNFL thickness and TMV.

It is believed that axonal loss in MS, in contrast to demyelination, is not reversible and may give rise to sustained disability. Despite the fact that axonal loss appears at final stages of MS, it can also be seen in its early stages.^19, 20^ On this account, in order to determine the disease development and provide appropriate therapeutic measures, timely appropriate monitoring of axonal loss is of crucial importance.^19^ Anterior optic pathways encompassing retina, optic nerve, optic chiasm, and optic tract are common places of axonal inflammation, demyelination, and degeneration.^21^ It was demonstrated that temporal-predominant peripapillary RNFL thinning is characteristic in MS; and the evidence for this RNFL thinning in clinical isolated syndrome (CIS) patients illustrates that temporal-predominant RNFL loss commences early in the course of MS.^22–24^ OCT rapidly produces high resolution images of retinal anatomy and appears to be useful in demonstrating global central nervous system pathology and disease process in MS.^5, 25^ It also seems that these findings show total intracranial volume in MS and normal subjects.^25^

Khanifar et al. studied RNFL in American subjects and observed a thinner RFNL in MS patients than in normal subjects. The authors also reported a significant association between RNFL thickness and disease duration. They found that patients with MS duration of more than five years represented the most conspicuous decline in RFNL thickness.^26^ These results corroborated our findings wherein the RFNL thickness mean in RRMS patients was lower than that of normal subjects. Findings of Khanifar et al. were inconsistent with those of the present study indicating the lowered RNFL thickness of each quadrant in the MS patients in comparison with the controls.

Fisher et al. investigated OCT findings in 90 patients suffering from MS and demonstrated that RNFL thickness notably declined in the MS patients with visual problems compared to controls. The decline was more marked in the MS patients presenting with ON.^15^ Bisaga et al. showed a marked decline in RNFL thickness in MS patients, especially those with ON, compared to healthy subjects.^27^ Herrero et al. studied progressive degeneration of RNFL in all quadrants and TMV in MS and normal subjects over 36 months. They stated that most of changes occurred in superior and inferior quadrants in the follow-up. The authors also argued that the patients who did not receive any treatment showed more degeneration in the superior RFNL quadrant.^28^ Fernandes et al. studied macular RNFL and retinal ganglion cell layer (RGCL) in 3 groups: 1) MS patients with ON; 2) MS patients without ON; and 3) patients with neuromyelitis optica in comparison with healthy controls. They reported diminished RFNL and RGCL thickness in all patients as compared to healthy subjects. However, no significant difference was demonstrated in RFNL and RGCL thickness between MS patients with ON and with neuromyelitis optica.^29^ Pulicken et al. also reported a thin RNFL in MS patients suffering from ON compared to healthy controls.^24^ Rebolleda et al. carried out a prospective study on RRMS and found, by means of stratus assessment, that MS patients presenting with ON showed decreased RNFL thickness in the temporal quadrant.^30^ Fatehi et al. in their study on patients with the definite history of optic neuritis regardless of the diagnosis of MS, found that there was a significant negative correlation between visual evoked potential (VEP) P100 latency and RNFL in all four quadrants. In addition, there was a significant correlation between P100 latencies and mean RNFL thickness. However, they found no significant difference in RNFL thickness between the three groups of CIS, RRMS, and secondary progressive MS patients.^31^

In the present study, amongst 60 MS patients, 35 cases presented with ON (19 cases with right eye involvement, 7 cases with left eye involvement, and 9 cases with both eyes’ involvement) and lowered RNFL thickness in all quadrants was associated with ON. Spain et al. studied the association of RNFL thickness with EDSS in 52 patients with MS. They found that the reduction in RNFL thickness was related to disease duration and EDSS. The authors also argued that reduced RNFL thickness and EDSS were separately associated with disease duration.^32^. In the current study, RNFL thickness in all quadrants of both eyes was observed to be associated with disease duration. Additionally, RNFL thickness in exclusively all the left eye quadrants and the nasal and temporal quadrants of the right eyes were seen to be associated with EDSS. It is worth noting that, unlike our study, in most preceding research the spectrum of patients did not exclusively include a specific type of RRMS and all four types were incorporated. Findings of the current study presented that macular volume decreased in patients with MS. Moreover, they showed that the mean for C/D ratio was less in MS patients than in controls.

To draw a conclusion, findings of the present study in the northwest of Iran buttress the idea that RNFL thickness can be greatly affected by MS. Our results also indicate that this effect is associated with ON, and MS duration and severity. These findings suggest that OCT findings can be utilized as a reliable marker for the follow-up during the course of the disease, and early detection of axonal damage and neuronal degeneration in MS, worldwide. Adding this approach to MS diagnostic protocols, thus, can provide valuable information in the diagnosis of MS.
